# Height as a Clinical Biomarker of Disease Burden in Adult Mitochondrial Disease

**DOI:** 10.1210/jc.2018-00957

**Published:** 2018-11-13

**Authors:** Rachel L Boal, Yi Shiau Ng, Sarah J Pickett, Andrew M Schaefer, Catherine Feeney, Alexandra Bright, Robert W Taylor, Doug M Turnbull, Grainne S Gorman, Tim Cheetham, Robert McFarland

**Affiliations:** 1 Department of Pediatric Endocrinology, Great North Children’s Hospital, Royal Victoria Infirmary, Newcastle Upon Tyne, United Kingdom; 2 Wellcome Centre for Mitochondrial Research, Institute of Neuroscience, Newcastle University, Newcastle Upon Tyne, United Kingdom; 3 Institute of Genetic Medicine, Newcastle University, Royal Victoria Infirmary, Newcastle Upon Tyne, United Kingdom

## Abstract

**Context:**

Abnormal growth and short stature are observed in patients with mitochondrial disease, but it is unclear whether there is a relationship between final adult height and disease severity.

**Objective:**

To determine whether patients with genetically confirmed mitochondrial disease are shorter than their peers and whether stature is related to disease severity.

**Design:**

Analysis of final adult height in relation to disease severity as determined by the Newcastle Mitochondrial Disease Adult Scale (NMDAS).

**Setting:**

UK Mitochondrial Disease Patient Cohort (Mito Cohort).

**Patients:**

575 patients were identified with recorded height, weight, and molecular genetic diagnosis of mitochondrial disease within the Mito Cohort.

**Main Outcome Measures:**

Adult height, body mass index (BMI), and their association with genetic subgroup and disease severity.

**Results:**

Adults with mitochondrial disease were short, with a mean height of −0.49 SD (95% CI, −0.58 to −0.39; n = 575) compared with UK reference data. Patients were overweight, with a BMI SD of 0.52 (95% CI, 0.37 to 0.67; n = 472). The most common genetic subgroup (m.3243A>G mutation) had a height SD of −0.70 (95% CI, −0.85 to −0.54; n = 234) and a BMI SD of 0.12 (95% CI, −0.10 to 0.34; n = 212). NMDAS scores were negatively correlated with height SD (*r* = −0.25; 95% CI, −0.33 to −0.17; *P* < 0.001, n = 533). Rate of disease progression also correlated negatively with adult height (*P* < 0.001).

**Conclusion:**

Final height in mitochondrial disease reflects disease severity and rate of disease progression. Mitochondrial dysfunction and associated subclinical comorbidities affect growth plate physiology.

## Teaser

Patients with mitochondrial disease are shorter than the general population, with adult height related to disease severity and rate of disease progression.

Mitochondrial disease has an estimated prevalence of 1 in 4300 adults (1). Disease can present with multisystem involvement, but neurologic features often predominate (2). Poor growth and short stature are well recognized in affected patients, yet the precise mechanisms resulting in growth failure are poorly understood.

Mitochondria are essential organelles found in all nucleated cells. Mitochondria have multiple roles in cellular metabolism, including a fundamental role in ATP synthesis through the process of oxidative phosphorylation. Mitochondria contain their own DNA [mitochondrial DNA (mtDNA)] encoding 37 genes. Thousands of copies of mtDNA may exist in an individual cell. One of several unique features of the mitochondrial genome is heteroplasmy, the coexistence of both mutated and wild type mtDNA within the same cell or tissue. Mutated mtDNA is often well tolerated at low levels of heteroplasmy, but once a specific threshold is exceeded, cellular and organ functions are disrupted (2). There are >1100 mitochondrial proteins encoded by nuclear genes, which are involved in the replication, translation, and maintenance of mtDNA, as well as other aspects of mitochondrial function (3). Mitochondrial disease can therefore arise as a consequence of a mutation in either mtDNA or the nuclear genome. In either case, the adverse impact on ATP generation is detrimental to organs with high energy demands such as the brain, heart, liver, and skeletal muscle.

The Newcastle Mitochondrial Disease Adult Scale (NMDAS) is a validated clinical scale used to assess multisystem involvement and disease progression. NMDAS explores all aspects of mitochondrial disease, focusing on current function and current clinical assessment, including system-specific involvement and quality of life (4). NMDAS is routinely performed on patients attending outpatient review by their neurologists in Newcastle Upon Tyne every 12 to 24 months. A low NMDAS score suggests the patient is asymptomatic or has low disease burden, whereas a high score reflects severe multisystem involvement.

The increase in height observed during childhood and adolescence is under the control of numerous factors, with a key physiological endpoint being growth and multiplication of growth plate chondrocytes (5). Mitochondrial dysfunction therefore has potential to undermine the growth process. Wolny *et al.* (6) reported that children with mitochondrial disease are about −1.97 SD shorter than their unaffected peers, with an associated BMI reduction of −1.07 SD (7). The m.3243A>G mutation in *MT-TL1* is the most common pathogenic mutation of mitochondrial disease and is commonly associated with short stature; 73% of a Chinese cohort of pediatric patients with this mutation were identified as being short (8). A similar observation was reported in a Japanese cohort, although a Dutch study found that short stature was observed only in female patients with mitochondrial disease (8–10). Patients with mitochondrial disease may not become symptomatic until adulthood, but they may still be shorter than expected, which suggests a subclinical effect the on growth plate metabolism.

We hypothesized that adults with mitochondrial disease would be shorter than their peers and that their stature would be related to disease severity and genetic subgroup.

## Subjects and Methods

We undertook a retrospective analysis of height, weight, and molecular genetic data from the UK Mitochondrial Disease Patient Cohort (Mito Cohort), collected from inception in March 2009 to February 2017. All patients provided informed consent when joining the Mito Cohort, a database of patients with genetically determined or biochemical evidence of mitochondrial disease. Data analysis was approved by the Mitochondrial Disease Cohort Oversight Committee. Height and body mass index (BMI) were converted to SDs based on UK reference data (British 1990 growth reference centiles) (11). The overall disease severity of individual patients was evaluated with the NMDAS, with the most recent score used in the analysis (4, 12).

We determined the relationship between final height and NMDAS scores in all adult patients where data were available. Additional analysis was conducted in specific groups of patients. They included patients with m.3243A>G, single large-scale mtDNA deletion, and nuclear gene defects. This analysis allowed us to determine whether there was any difference in the association of height, BMI, and NMDAS between genotypes. The m.3243A>G mutation is the single most common cause of mitochondrial disease (1). In patients with m.3243A>G mutation, age-adjusted blood heteroplasmy levels were also analyzed (13). In the single, large-scale mtDNA deletion group, we performed additional analysis by looking at Kearns-Sayre syndrome (KSS), chronic progressive external ophthalmoplegia and ptosis (CPEO), and CPEO plus (CPEO+) because those with KSS are known to have early-onset disease, whereas those with CPEO and CPEO+ present later in life (2).

Normality testing was performed before statistical analysis, with nonnormally distributed data subject to square root transformation before parametric testing. The square root of NMDAS score was therefore included in all described analyses (13). The independent samples *t* test was used to compare means between two groups. ANOVA was used to compare means between multiple groups; subsequent comparisons between groups were evaluated with Bonferroni-corrected *t* tests. The association of two continuous variables was tested via Pearson correlation. All means are displayed with 95% CIs unless otherwise stated. The statistical significance threshold was set at *P* < 0.05 for all tests.

We determined the relationship between final adult height and BMI between disease manifestations as defined by questions in the NMDAS score with Pearson correlation. To quantify the association between height SD, BMI SD, age, and progression of disease burden, we used the Linear and Nonlinear Mixed Effects Models package to perform a linear mixed effects analysis of the relationship between heteroplasmy level, age, and NMDAS scaled score (14). As fixed effects we entered age and height SD or BMI SD into the model. As random effects we had by-subject random slopes for the effect of age. Two-sided *P* values were obtained from the *t* distribution reported in Linear and Nonlinear Mixed Effects Models.

In our cohort we are not aware of any patients with mitochondrial disease who have been treated with GH. Short stature due to mitochondrial disease is not an indication for GH treatment in the United Kingdom (15).

## Results

A total of 891 participants were enrolled in the Mito Cohort in Newcastle, of whom 797 patients were aged ≥18 years by January 2017. A total of 222 patients were excluded because of a lack of recorded height (n = 143), no established genetic cause (n = 78), or a height data entry error (n = 1). A total of 575 adults aged 18 to 85 years at the time of their height measurement, were identified with genetic evidence of mitochondrial disease and growth data available (Table 1). Of these, 38.3% were male (n = 220) and 61.3% female (n = 355).

**Table 1.** Cohort Demographics

Genetic ID	Genetic Diagnosis	Number of Patients	Male	Female	Mean Age at Height Measurement	Age Range	Mean Height SD	95% CI of the Mean for Height SD	Number of Patients With BMI	Mean BMI SD	95% CI of the Mean for BMI SD
All cohort		575	220	355	46	18–85	−0.49	−0.58 to −0.39	472	0.52	0.37 to 0.67
Primary mtDNA mutations											
	m.14709T>C	11	1	10	48	26–64	0.30	−0.57 to 1.17	7	−0.24	−3.00 to 2.53
	m.3243A>G	234	95	139	42	18–79	−0.70	−0.85 to −0.55	212	0.12	−0.10 to 0.34
	m.8344A>G	40	13	27	45	20–69	−0.41	−0.72 to −0.11	28	0.66	−0.01 to 1.34
	Single large-scale mtDNA deletion	90	34	56	47	18–85	−0.27	−0.52 to −0.03	78	0.68	0.33 to 1.03
	Other^*a*^	54	20	34	42	20–77	−0.33	−0.59 to −0.06	40	0.72	0.23 to 1.20
Nuclear DNA mutations											
	*OPA1*	17	5	12	46	18–72	−0.28	−0.91 to 0.35	13	2.11	1.56 to 2.67
	*POLG*	27	11	16	49	19–80	−0.09	−0.50 to 0.31	19	1.00	−0.03 to 1.96
	*RRM2B*	17	5	12	59	31–76	−0.30	−0.88 to 0.29	14	0.97	0.34 to 1.60
	*TWINK*	40	15	25	58	18–82	−0.42	−0.73 to −0.11	32	1.32	0.79 to 1.85
	Other multiple deletion	27	12	15	63	45–77	−0.69	−1.22 to −0.16	15	1.27	0.64 to 1.91
	Other^*b*^	18	9	9	39	18–81	−0.66	−1.24 to −0.09	14	0.00	−1.60 to 1.61

^a^ Other primary mtDNA mutations include m.11778G>A, m.12147G>A, m.12148T>C, m.12258C>A, m.12271T>C, m.12283A>G, m.12315G>A, m.12320A>G, m.13051A>G, m.13094T>C, m.13513G>A, m.14674T>C, m.15699G>C, m.16002T>C, m.16023G>A, m.1624C>T, m.3243A>T, m.3365T>C, m.3460G>A, m.4175G>A, m.4267A>G, m.4298G>A, m.4300A>G, m.5543T>C, m.5650A>G, m.5690A>G, m.618T>G, m.7497G>A, m.7587T>C, m.7989T>C, m.8839G>C, m.8851T>C, m.8993T>C, m.8993T>G, m.9176T>C, and m.9185T>C.

^b^ Other nuclear DNA mutations include the following: *AFG3L2* encodes AFG3-like protein 2, *ETFDH* encodes electron transfer flavoprotein dehydrogenase, *GFER* encodes growth factor augmenter of liver regeneration, *MTFMT* encodes mitochondrial methionyl-tRNA formyltransferase, *OPA1* encodes dynamin-like 120-kDa protein, *PDH* encodes pyruvate dehydrogenase complex, *POLG* encodes the α-subunit of mitochondrial polymerase-γ, *RRM2B* encodes ribonucleotide reductase regulatory TP53 inducible subunit M2B, *SDHA* encodes succinate dehydrogenase complex subunit A, *SPG7* encodes paraplegin, *TRIT1* encodes tRNA isopentenyltransferase, *TWNK* (previously also known as *PEO1*) encodes twinkle helicase, *TYMP* encodes thymidine phosphorylase, and *YARS2* encodes tryosyl-tRNA synthetase.

The height recorded at ≥18 years of age was assumed to be the patient’s final height. In 38 patients, the date of height measurement was not documented; for these patients final height was assumed to be their consent date for entry in the cohort, provided they were >18 years old.

Subsequent analysis was performed on all patients and specific heterogeneous groups: m.3242A>G (n = 234), nuclear gene defects (n = 146), and single large-scale mtDNA deletions (n = 90). We omitted other mtDNA mutations (n = 107) because the sample size for each genotype was insufficient.

### Age

The mean age at height measurement of patients recruited to the cohort was 46.1 years (95% CI, 44.8 to 47.5; n = 538). The date height was taken was not available for 38 patients (Table 1). The mean age at height measurement in the m.3243A>G group was 42.4 years (95% CI, 40.6 to 44.3; n = 226), in the single large-scale deletion group it was 47.3 years (95% CI, 44.0 to 50.6; n = 84), and in the nuclear gene defect group it was 53.5 years (95% CI, 50.5 to 56.5; n = 129).

There was a significant difference between mean age at height measurement between the genetic subgroups, as determined by one-way ANOVA [F(2, 436) = 21.38, *P* < 0.001]. A multiple comparison, using Bonferroni corrected *t* tests, revealed that the mean age at height measurement was significantly lower in the m.3243A>G group than in the nuclear gene defect group (mean difference = −11.09; 95% CI, −15.17 to −7.00; *P* < 0.001) and the single large scale deletion group (mean difference = −4.85; 95% CI, −9.58 to −0.12; *P* = 0.042). There was also a significant difference in the age at height measurement between the single large-scale deletion group and nuclear groups (mean difference = −6.23; 95% CI, −11.42 to −1.05; *P* = 0.012).

### Height

**Figure 1.**
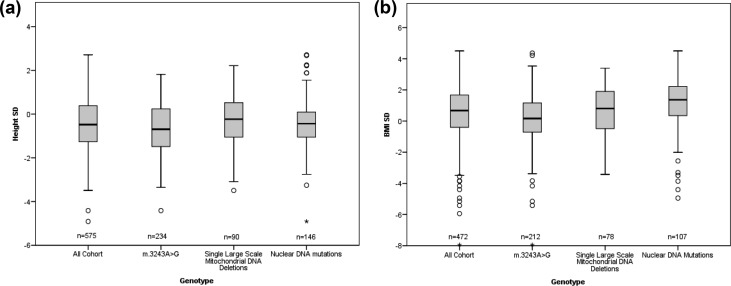
Individual boxplots of height SD and BMI SD for genotypes. Boxplots showing the median, first quartile, third quartile, and range for (a) height SD and (b) BMI SD for the cohort and each genotype.

There was no significant difference in the mean height SD score (SDS) between men (mean = −0.42; SD 1.13; 95% CI, −0.57 to −0.27; n = 220) and women [mean = −0.52; SD 1.18; 95% CI, −0.65 to −0.40; n = 335; t(573) = 1.02; *P* = 0.307; 95% CI, −0.94 to 0.29]. The 575 adults with mitochondrial disease were shorter than the general population, with a mean SD for height of −0.49 (95% CI, −0.58 to −0.39) (Table 1, Fig. 1). Furthermore, 34.6% (n = 199) of adults had a height >1 SD below the mean, and 10% (n = 57) of adults had a height >2 SD below the mean.

The mean height SD of the m.3243A>G subgroup was −0.70 (95% CI, −0.85 to −0.54; n = 234), in the single large-scale deletion group it was −0.27 (95% CI, −0.52 to −0.03; n = 90), and in the nuclear group it was −0.41 (95% CI, −0.59 to −0.22; n = 146). There was a significant difference in mean final height SDs between genetic subgroups as determined by one-way ANOVA [*F*(2, 467) = 5.37, *P* = 0.005]. Multiple comparison (Bonferroni corrected) revealed that the mean final height SD was significantly lower in the m.3243A>G group than in the single large-scale deletion group (mean difference = −0.43; 95% CI, −0.77 to −0.08; *P* = 0.011). There was no difference between the m.3243A>G and nuclear groups (mean difference = −0.29; 95% CI, −0.59 to 0.01; *P* = 0.057). There was no significant difference between the single large-scale deletion group and the nuclear deletion group (mean difference = 0.13; 95% CI, −0.51 to 0.24; *P* = 1.000). In the m.3243A>G group, 42% (n = 102) had a height of <1 SD below the UK reference data, and 15% (n = 36) had a height <2 SD below the UK reference data.

There is limited data on when patients with mitochondrial disease attain their final height and whether this timing is delayed. In our cohort, final adult height was not significantly lower in the patients whose heights were recorded between 18 and 25 years of age (mean = −0.73; SD 1.32; 95% CI, −1.09 to −0.38; n = 55) compared with those where height was recorded at >25 years of age [mean = −0.46; SD 1.10; 95% CI, −0.56 to −0.36; n = 482; t(535) = −1.69; *P* = 0.092; 95% CI, −0.59 to −0.64]. This equates to a mean height of 159.2 cm for women and 172.5 cm for men <25 years and mean height of 160.9 cm for women and 174.1 cm for men >25 years.

In an analysis of the single large-scale mtDNA deletion, patients with KSS had a mean height SD of −1.41 (95% CI, −2.31 to −0.51; n = 11), patients with CPEO had a mean height SD of 0.33 (95% CI, −0.15 to −0.81; n = 20), and those with CPEO+ had a mean height SD of −0.28 (95% CI, −0.59 to 0.02; n = 50).

### BMI

In total, 82.1% (n = 472) of patients had a recorded weight, which was used to calculate BMI SD. The mean weight in men (75.4 kg; 95% CI, 72.6 to 78.3; n = 182) was higher than in women (63.6 kg; 95% CI, 61.7 to 65.5; n = 290). There was no significant difference in BMI SD between men (mean = 0.48, SD 1.84, n = 182) and women [mean = 0.54, SD 1.62, n = 290; t(470) = −0.37; *P* = 0.713; 95% CI, −0.353 to 0.288] (Table 1).

A mean BMI SD of 0.52 (95% CI, 0.37 to 0.67; n = 472) was above average when compared with UK standards [Fig. 1(b)]. The mean BMI SD for the m.3243A>G group was 0.12 (95% CI, −0.10 to 0.34; n = 212), for the single large-scale deletion group it was 0.68 (95% CI, 0.33 to 1.03; n = 78), and for the nuclear group it was 1.13 (95% CI, 0.80 to 1.46; n = 107). There was an observed difference in mean BMI SD [*F*(2, 394) = 14.04, *P* < 0.001] between the genetic subgroups. Multiple comparisons revealed that the m.3243A>G subgroup had a lower mean BMI SD than the single large-scale deletion group (mean difference = −0.55; 95% CI, −1.08 to −0.04; *P* = 0.032) and the nuclear group (mean difference = −1.01; 95% CI, −1.48 to −0.54; *P* < 0.001).

Further analysis of single, large-scale mtDNA deletion data showed that patients with KSS had a mean BMI SD of −0.55 (95% CI, −2.02 to 0.92; n = 9), patients with CPEO had a mean BMI SD of 1.02 (95% CI, 0.31 to 1.74; n = 18), and those with CPEO+ a mean BMI SD of 0.78 (95% CI, 0.35 to 0.78; n = 46).

### NMDAS

**Figure 2.**
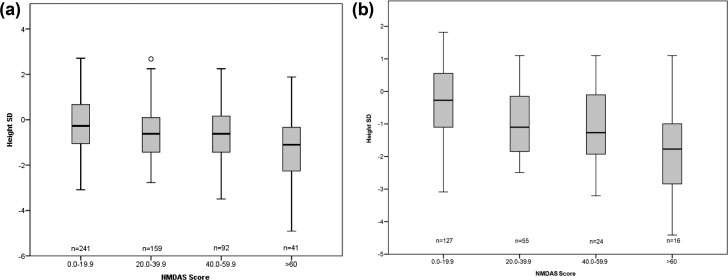
Individual boxplots of height SD against NMDAS score. Boxplots showing the median, first quartile, third quartile, and range for height SD (a) all cohort and (b) m.3243A>G against NMDAS score. All cohort n = 533, and m.3243A>G n = 222.

A total of 533 adults had a documented height and NMDAS score. NMDAS scores were negatively correlated with height SDS (Pearson correlation coefficient *r* = −0.25; 95% CI, −0.33 to −0.17; *P* < 0.001; n = 533) (Fig. 2). A weaker correlation was observed between the NMDAS score and BMI SD (*r* = −0.10; 95% CI, −0.19 to −0.01; *P* = 0.033; n = 439).

In the m.3243A>G group the NMDAS score was negatively correlated with the height SD (*r* = −0.39; 95% CI, −0.50 to −0.27; *P* < 0.001; n = 222). The NMDAS score was also negatively correlated with BMI SDS in this subgroup (*r* = −0.32; 95% CI, −0.44 to −0.20; *P* < 0.001; n = 201). In the single, large-scale mtDNA deletion group the NMDAS score was negatively correlated with height SDS (*r* = −0.30; 95% CI, −0.47 to −0.12; *P* = 0.006; n = 82); however, there was no association between BMI and NMDAS score (*r* = −0.18; 95% CI, −0.37 to 0.043; *P* = 0.130; n = 72). In the nuclear deletion group the NMDAS score was negatively correlated with height SDS (*r* = −0.193; 95% CI, −0.34 to −0.03; *P* = 0.027; n = 131). There was no correlation between BMI and NMDAS score (*r* = 0.12; 95% CI, −0.06 to 0.30; *P* = 0.262; n = 96).

Mean NMDAS score of those <25 years of age when height was recorded was 23 (95% CI, 16.3 to 29.9; n = 46), and the NMDAS score of those >25 years of age when height was recorded was 26 (95% CI, 24.6 to 28.1; n = 453). NMDAS score was negatively correlated with the height SD of patients <25 years of age when height was recorded (*r* = −0.43; 95% CI, −0.63 to −0.18; *P* = 0.003; n = 46) and >25 years when height was recorded (*r* = −0.22; 95% CI, −0.31 to −0.14; *P* < 0.001; n = 453).

### Heteroplasmy

Age-corrected blood mutant heteroplasmy was explored in our largest subgroup, m.3243A>G (13). Data were available for 92.3% (n = 216) of patients with m.3243A>G. NMDAS score was positively correlated with age-corrected blood heteroplasmy level (*r* = 0.36; 95% CI, 0.28 to 0.47, *P* < 0.001). The age-corrected blood heteroplasmy level was negatively correlated with height (*r* = −0.31; 95% CI, −0.42 to −0.18; *P* < 0.001) and BMI (*r* = −0.39; 95% CI, −0.50 to −0.26, *P* < 0.001) SDs.

### Patient-reported disease onset

Further information with regard to patient-reported symptom onset was explored in the two largest subgroups with the m.3243A>G mutation and single large-scale mtDNA deletions. In the m.3243A>G group, onset data were available for 86.8% (n = 166) of patients, of whom 18.2% (n = 37) were asymptomatic carriers. The mean age of onset of patients in the m.3243A>G group was 28.5 years (95% CI, 26.5 to 30.6; n = 166). Age of onset was available for 96.7% (n = 87) of patients with single large-scale deletions, and the mean age of onset was 23.7 years (95% CI, 21.07 to 26.47; n = 87).

In the m.3243A>G mutation group a positive correlation was identified between age of onset and height SD (*r* = 0.28; 95% CI, 0.13 to 0.41; *P* ≤ 0.001; n = 166), indicating that later onset was associated with taller final adult height. A positive correlation was also identified between age of onset and BMI SD (*r* = 0.27; 95% CI, 0.12 to 0.41; *P* ≤ 0.001; n = 155). No correlation was identified between NMDAS score and age of onset (*r* = −0.10; 95% CI, −0.25 to 0.06; *P* = 0.220; n = 159).

In the single, large-scale mtDNA deletion group no relationship was observed between age of onset and final adult height SD (*r* = 0.010; 95% CI, −0.20 to 0.22; *P* = 0.912; n = 87), BMI SD (*r* = 0.05; 95% CI, −0.18. to 0.27; *P* = 0.678; n = 77), or NMDAS score (*r* = −0.17; 95% CI, −0.37 to 0.07; *P* = 0.136; n = 80). In patients with KSS, the mean age of onset was earlier, at 11.1 (±6.2) years of age, compared with other genetic groups.

### Disease manifestations and progression

There were 400 patients with multiple NMDAS measurements: 174 in the m.3243A>G subgroup, 90 in the nuclear group, 71 with single deletions, and 65 in the other subgroup. The median number of NMDAS assessments was 4 (interquartile range = 4.00; range = 2 to 22), with a median interval between NMDAS assessments of 5.20 years (interquartile range = 5.85; range = 0, 13.3). Longitudinal linear mixed modeling, used to examine the role of height and BMI in the rate of NMDAS development, revealed a significant negative association between height SD and increased disease progression in the whole cohort (*P* ≤ 0.001). This association was driven mainly by the m.3243A>G subgroup (*P* ≤ 0.001). The nuclear gene defects and single large-scale mtDNA deletion groups showed no significant association (*P* = 0.197 and *P* = 0.144, respectively).

A similar picture was seen when BMI SD was considered. The significant association with increased disease progression in the whole cohort (*P* ≤ 0.001) was driven mainly by the m.3243A>G subgroup (*P* ≤ 0.001), although the single large-scale mtDNA deletion subgroup also showed a significant effect (*P* = 0.021). The nuclear subgroups showed no association (*P* = 0.188).

Height SD was negatively correlated with several specific NMDAS fields, namely cerebellar ataxia, cognition, pyramidal involvement, hearing impairment, gastrointestinal disturbance, diabetes, and cardiovascular involvement (range of *r* = −0.17 to −0.28, all *P* < 0.001; Table 2). Interestingly, although hearing impairment and gastrointestinal disturbance are also associated with BMI SD (*r* for both = −0.17, *P* < 0.001), BMI SD is independently associated with four other phenotypes not associated with height SD (dysphonia or dysarthria, seizures, encephalopathy, and strokelike episodes; range of *r* is −0.16 to 0.21, *P* < 0.001).

**Table 2.** Correlation of Height and BMI SDs With Disease Manifestations

NMDAS Question	Height SD				BMI SD			
	*r*	95% CI	*P*	n	*r*	95% CI	*P*	n
Psychiatric involvement	−0.10	−0.19 to −0.02	0.016	545	−0.06	−0.15 to 0.03	0.193	447
Cerebellar ataxia	−0.20	−0.28 to −0.11	<0.001^*a*^	536	−0.06	−0.15 to 0.04	0.238	442
Migraine	−0.10	−0.18 to −0.01	0.026	545	−0.10	−0.19 to 0.00	0.039	447
Cognition	−0.28	−0.36 to −0.20	<0.001^*a*^	515	−0.13	−0.22 to −0.04	0.007	426
Neuropathy	−0.01	−0.10 to 0.07	0.785	538	0.07	−0.02 to 0.16	0.128	443
Dysphonia or dysarthria	−0.13	−0.22 to −0.05	0.002	539	−0.16	−0.25 to −0.07	0.001^*a*^	442
Seizures	−0.08	−0.16 to 0.00	0.065	545	−0.21	−0.30 to −0.12	<0.001^*a*^	447
Encephalopathy	−0.09	−0.18 to −0.01	0.031	545	−0.22	−0.31 to −0.13	<0.001^*a*^	447
Strokelike episodes	−0.10	−0.18 to −0.01	0.025	545	−0.22	−0.31 to −0.13	<0.001^*a*^	447
Pyramidal	−0.19	−0.27 to −0.11	<0.001^*a*^	537	−0.11	−0.20 to −0.02	0.017	443
Extrapyramidal	−0.13	−0.21 to −0.05	0.002	537	−0.02	−0.12 to 0.07	0.604	443
Visual acuity	−0.12	−0.21 to −0.04	0.005	529	0.09	0.00 to 0.19	0.049	435
Ptosis	−0.01	−0.10 to 0.07	0.747	539	0.08	−0.01 to 0.18	0.075	443
CPEO	0.02	−0.07 to 0.10	0.701	540	0.05	−0.04 to 0.14	0.288	443
Hearing impairment	−0.24	−0.32 to −0.16	<0.001^*a*^	545	−0.17	−0.26 to −0.08	<0.001^*a*^	447
Gastrointestinal disturbance	−0.17	−0.25 to −0.08	<0.001^*a*^	545	−0.17	−0.26 to −0.08	<0.001^*a*^	447
Myopathy	−0.07	−0.15 to 0.02	0.119	540	−0.10	−0.19 to −0.01	0.031	443
Diabetes	−0.19	−0.27 to −0.10	<0.001^*a*^	545	0.02	−0.07 to 0.11	0.649	447
Cardiovascular involvement	−0.21	−0.29 to −0.12	<0.001^*a*^	495	−0.07	−0.16 to 0.03	0.170	414

Pearson correlations of height SD and BMI SD with comorbidities as defined by individual NMDAS questions. Estimated Pearson correlation coefficients and uncorrected *P* values are shown. The Bonferroni corrected threshold of significance for correlations is 0.001 because 38 independent tests were performed. Disease manifestations represent answers to questions from sections II and III of the NMDAS assessment, because these are physician assessed and therefore more likely to be objective. Hearing impairment from section I was included, because this is the only assessment of this common mitochondrial disease manifestation within the NMDAS.

^a^ Significant results.

## Discussion

Our findings in a large cohort of well-characterized patients supports earlier work indicating that patients with mitochondrial disease are shorter than their peers. In addition, we describe associations between disease severity as measured by the NMDAS and rate of disease progression with reduced final adult height. We have also noted that disease severity is associated with nutritional status, as determined by BMI, in patients with the m.3243A>G mutation, although the overall BMI SD of this patient group is well within normal limits.

It was interesting to note that patients with short stature were more likely to have diabetes and cardiovascular involvement, whereas patients with a low BMI had more severe symptoms such as seizures, encephalopathy, and strokelike episodes, as noted previously (16). Both short stature and low BMI were associated with hearing impairment and gastrointestinal disturbances.

Because adults often attain their final height before the onset of overt disease, our findings suggest that otherwise subclinical impairments in mitochondrial function can affect growth and specifically the behavior of the chondrocyte.

There are a number of possible explanations for the association between phenotype and stature. Muscle function is known to affect skeletal integrity and bone mass, but our data may reflect an impact of mitochondrial and associated muscle dysfunction on bone growth. With this in mind, it is of interest that patients with the milder Becker muscular dystrophy phenotype are taller than those with Duchenne muscular dystrophy, and in children with cerebral palsy, more profound motor dysfunction is associated with slower growth (17, 18).

Increasing evidence in animal models shows the importance of muscle contractions in longitudinal bone growth; in one study mice with one limb paralyzed had a significantly shorter limb length in the affected limb, demonstrating that muscle loading has a role in stimulating chondrocytes in the growth plate (19).

Although it is possible that reduced muscle mass and exercise intolerance during childhood in patients with early onset mitochondrial disease could affect the growth plate, a more direct effect of abnormal mitochondrial function on chondrocyte function can also be postulated. Hence, dysfunctional ATP synthesis within the chondrocyte itself could be a mechanism leading to poor growth and short stature. In the growth plate, hypertrophy and proliferation of chondrocytes are closely regulated, and it is well recognized that in these cells decreased ATP results in apoptosis (19). In patients with mitochondrial disease this effect may result in increased apoptosis and cell death in the growth plate, with associated suboptimal growth. An increase in apoptosis has been observed in the muscle samples of patients with primary mitochondrial disorders, and a similar process may affect the chondrocyte as a potential mechanism (20).

Another potential explanation for poor growth in these patients is that abnormal mitochondrial function affects GH production and secretion. There have been numerous case reports of GH deficiency in patients with mitochondrial disease in the nuclear, m.3243A>G, and single large-scale mtDNA deletions (21–24). Postulated mechanisms include the presence of mutated mtDNA in the hypophysis, leading to pituitary dysfunction (21, 25).

Primary lesions affecting the hypothalamic GH releasing factor cells have been reported in two patients with mitochondrial encephalomyopathy lactic acidosis and strokelike episode, with increased GH production noted after the administration of GH releasing factor (23). Treatment with recombinant GH improved stature in a small number of patients with mitochondrial disease, but there are no larger-scale studies (23, 26, 27). Concerns have been raised about possible long-term detrimental effects of increased GH concentrations in mitochondrial disease, with acute deteriorations reported in patients with KSS and other mitochondrial rearrangements due to increased tissue energy demands after administration (22, 26, 27).

Suboptimal nutrition and an inability to meet metabolic energy demands could also influence stature and body habitus, although overall BMI SD was above average. Gastrointestinal symptoms commonly reported by patients with mitochondrial disease include reduced oral intake due to poor appetite and early satiety. Dysphagia of varying severity is also well recognized, affecting 48% of patients and is very common in the m.3243A>G subgroup (9, 28). The mechanism is believed to be dysfunctional involvement of smooth muscle, peripheral nerves, and central nervous system in swallowing. Gastroparesis, delayed gastric emptying, bacterial overgrowth, and intestinal pseudo-obstruction have also been noted in this population (16). Malabsorption is commonly experienced by patients, and mtDNA defects have been shown to impair human colonic epithelial function (29). Together, these mechanisms result in malabsorption and reduced body mass. Our data also demonstrate that there is an association between disease severity and BMI. In the m.3243A>G subgroup, disease severity as measured by NMDAS is associated with a fall in BMI. We also noted that patients with KSS, known for their early onset of disease manifestations, are shorter, with a lower BMI than those with CPEO, whose onset is often later in life (30). Body mass and muscle strength affect mechanical loading, which is essential for bone remodeling and maintaining mineral density. Those with a higher BMI have a greater bone mineral density because of mechanical loading (9, 31).

As well as gastrointestinal symptoms, patients with mitochondrial disease may have other comorbidities that could theoretically affect growth and bone health (9, 32, 33). These include endocrinopathies such as hypoparathyroidism, hypothyroidism, and diabetes. However, these endocrine disorders are variably expressed within patients who have the same genotype. Indeed, phenotypic heterogeneity and tissue-specific vulnerability are often observed across both mtDNA and nuclear gene defects. In this study, our finding that patients with mitochondrial disease caused by the m.3243A>G mutation are generally shorter than patients with other genetic mutations is entirely consistent with this phenotypic heterogeneity, but the exact mechanism of such tissue-specific changes remains elusive.

There is a growing understanding that the systemic effects of stress and inflammation in chronic disease states may affect growth (34). In adults with mitochondrial disease, hypothyroidism is uncommon, and mitochondrial diabetes usually develops after the age of 35 years and hence beyond the phase of childhood and adolescence when growth occurs (35, 36). Hypoparathyroidism is seen in severe childhood-onset KSS and mitochondrial encephalomyopathy lactic acidosis and strokelike episode, but in these patients short stature is caused by multisystem involvement. Patients with severe mitochondrial disease are more likely to be receiving multiple medications, which may also affect growth; however, because the majority of our patients attained final height before overt disease onset, this potential mechanism is unlikely. The prevalence of one risk factor affecting bone health in mitochondrial disease was estimated at 73% in one cohort, with 30% having four risk factors (33). Patients with mitochondrial disease are at elevated risk of osteoporosis, with more than half of the adult population known to have at least one risk factor (31).

Multiple comorbidities and associated stress could result in elevated circulating levels of growth differentiation factor 15 (37). growth differentiation factor 15 has been cited as a possible biomarker of mitochondrial disease (38, 39). It inhibits the production of IGF-1 in the liver, limiting body size in response to decreased energy availability. This pathophysiological process could affect growth during childhood and adolescence in the context of patients subsequently diagnosed with a mitochondrial disorder.

### Limitations

Missing data in height, weight, and NMDAS scores in some patients may result in selection bias. However, there was no significant difference between the mean NMDAS score of our cohort (n = 537) and those excluded for having no documented height (n = 36) (27 vs 33, *P* = 0.096).

We wondered whether the negative association between NMDAS score and stature might reflect the impact of the disease process on progress through puberty with a recorded height that was not representative of final adult height in younger patients. An analysis of the association between NMDAS score and height in older patients was similar to the data as a whole, confirming that the association was not simply caused by delayed pubertal development and growth. At present, limited information is available about growth in patients with mitochondrial disease during childhood and adolescence.

Stature is multifactorial, and in the pediatric population midparental height is often used by clinicians to determine a child’s potential final adult height and to look for a possible growth disorder. In our cohort no parental final height data were available; it would be useful to consider whether stature in those with a mitochondrial defect differed from that of unaffected parents.

Intrauterine growth is known to affect final height. In our cohort birthweight and gestation data were not available. Studies looking at birth weight in mitochondrial disease have focused on patients with pediatric mitochondrial disease and have shown that they are significantly lighter than their counterparts; in one study ≤22% were below the third percentile for weight at birth (40, 41). We know that infants who are small for gestational age often attain a shorter final height than their larger counterparts, particularly if no catch-up growth is demonstrated within the first years of life. Our pregnancy study has recently shown that lower birth weight was evident in the m.3243A>G group compared with other genotypes. It is therefore possible that short stature in patients with more severe mitochondrial disease is linked to smaller size at birth, and this is an area that requires further study.

Because our data are predominantly single-point data, the impact of loss of vertebral height due to osteoporosis and aging is important to consider. We addressed this limitation by correlating age at height measurement and height SDs and found no significant correlation (*r* = −0.01; 95% CI, −0.10 to 0.07; *P* = 0.80; n = 537). Nevertheless, it is possible that current height is less than the maximally attained height in a small number of older patients who have osteoporotic fracture, causing loss of vertebral height, or simply the normal age-related change in adult height.

## Conclusions

This is a comprehensive description of stature in adults with mitochondrial disease. Numerous mechanisms for short stature can be postulated, including the impact of muscle function on the growth plate, abnormal chondrocyte activity and suboptimal nutrition. To date the definitive mechanism or mechanisms remain unknown.

We suspect that along with the impact of chronic disease, abnormalities of growth in mitochondrial disease also reflect abnormal chondrocyte behavior within the growth plate. More animal mechanistic studies are needed to confirm this suspicion. The role of heteroplasmy levels in chondrocytes remains to be explored.

Our findings suggest that height is a simple measure that may have a predictive value for the disease burden in adult mitochondrial disease. Additional observational analysis of serial measurements of NMDAS scores, height, and weight would help explore these associations in greater detail. Accurate growth data may help to guide clinicians and families toward the more appropriate use of targeted physiotherapy programs in the future. Short stature may be an early indicator of mitochondrial disease and should be considered within the differential diagnosis to optimize patient care.

## Abbreviations:

BMI: body mass index
CPEO: chronic progressive external ophthalmoplegia and ptosis
CPEO+: chronic progressive external ophthalmoplegia and ptosis plus
KSS: Kearns-Sayre syndrome
Mito Cohort: UK Mitochondrial Disease Patient Cohort
mtDNA: mitochondrial DNA
NMDAS: Newcastle Mitochondrial Disease Adult Scale
SDS: standard deviation score
